# Genomic insights into probiotic functionality of *Enterococcus hirae* 3K isolated from Egyptian coastal sediments with special reference to exopolysaccharide production and antimicrobial activity potential

**DOI:** 10.1186/s12866-026-04830-1

**Published:** 2026-03-05

**Authors:** Mennat Allah A. Lamada, Nancy M. El Halfawy, Amira M. Hamdan, Moustafa Y. El-Naggar

**Affiliations:** 1 Botany and Microbiology Department, Faculty of Science, Alexandria University, Alexandria, Egypt; 2 Oceanography Department, Faculty of Science, Alexandria University, Alexandria, Egypt

**Keywords:** *Enterococcus hirae*, Exopolysaccharide, Probiotic, Whole genome sequence (WGS), Safety assessment.

## Abstract

Marine lactic acid bacteria (LAB) are beneficial microbes that play a vital role in maintaining aquatic ecosystems and have significant potential for developing innovative products. Notably, marine LAB represent promising probiotic candidates due to their high efficacy and adaptability. In this study, strain 3K was isolated from coastal sediments in Egypt. Phenotypically, isolate 3K strain was susceptible to most antibiotics assessed, exhibited tolerance to low pH and high bile salts conditions, and revealed the absence of hemolytic activity. Furthermore, whole genome sequencing (WGS) was conducted using the Illumina NovaSeq 6000 platform with 60X coverage to assess the genomic probiotic attributes of marine strain 3K. In addition, the genome size of the *Enterococcus hirae* 3K strain was 2.91 Mb, with a G + C content of 36.65%. Genomic analysis confirmed the absence of critical virulence-factor determinants in the sequenced genome. Nevertheless, vancomycin resistance determinants were not identified in the genome sequence. Genome mining identified genes encoding enterolysin A and lanthipeptide bacteriocins, signifying a broad antimicrobial potential that correlates with the observed inhibitory activity against the target microbial pathogens. Additionally, exopolysaccharide (EPS) biosynthetic clusters were also predicted in silico, indicating the capacity for EPS production. Furthermore, the comparative genomic analysis revealed 2,216 shared orthologous clusters, confirming species-level conservation. The findings of WGS and phenotypic analyses confirmed the safety, metabolic versatility, and probiotic potential of the marine *E. hirae* 3K. Notably, its isolation from marine sediment highlights an underexplored ecological niche, reinforcing its potential as a health-promoting probiotic candidate with distinct biotechnological relevance for sustainable aquaculture applications.

**Supplementary Information:**

The online version contains supplementary material available at 10.1186/s12866-026-04830-1.

## Background

Marine lactic acid bacteria (LAB) function as probiotic reservoirs, comprising advantageous microorganisms that inhabit complex ecosystems. Within these environments, they are exposed to physiologically challenging conditions and, through adaptive mechanisms, have evolved to thrive under harsh environmental stresses [1, 2]. They are gaining much attention for their potential applications in boosting marine health [3]. Also, they can synthesize a wide range of secondary metabolites and bioactive substances with unique properties to adapt to these unique environmental conditions [2]. Moreover, marine probiotics producers can be isolated from sponges, seaweeds, and fish in the marine environments, and their by-products play a significant role in multiple industrial sectors, notably in food processing, fermentation technology, pharmaceutical, and biopolymer industries [4].

Selecting the appropriate probiotic strains and determining their effective dosages are critical parameters for eliciting targeted health benefits [5]. Genome mining facilitates the assessment of genetic stability, the identification of probiotic-associated genes, and the discovery of biosynthetic pathways for vitamins, exopolysaccharides (EPS), and bioactive compounds with potential health-promoting effects [6]. A comprehensive assessment of potential risks associated with probiotic strains remains a necessary component of their preclinical evaluation. Therefore, employing whole genome sequencing (WGS) technology enables the determination of virulence, antibiotic resistance (AMR), and toxin genes [7]. The integration of these advanced technologies enables the systematic identification and selection of targeted strains possessing significant industrial value.

*Enterococcus hirae* is one of the predominant bacterial species identified within marine sediments [8, 9]. *E. hirae* serve as a crucial source for promising probiotic candidates, frequently isolated from aquatic hosts, for example, fish, shrimps, and fermented food matrices [10]. For instance, *E. hirae* F2 demonstrated broad-spectrum antagonistic activity against pathogenic indicators, including *Escherichia coli*, *Staphylococcus aureus*, *Salmonella Typhimurium*, and *Pseudomonas* sp [11]. *E. hirae* MLG3-25-1 emphasized its role as a probiotic strain against *Bacillus* sp. by producing bioactive compounds like EPS [12]. However, genomic data for *E. hirae* strains of marine sediment origin are particularly scarce. Addressing this limitation through comprehensive genomic analysis is crucial to reveal their unique functional and adaptive profiles.

Hence, this study aimed to characterize the *E. hirae* 3K strain isolated from marine sediments, with particular emphasis on its probiotic and biotechnological potential. The objectives were to evaluate strain safety through comparative phenotypic and antibiotic susceptibility analyses, assess genomic features using whole-genome sequencing and functional annotation, and determine the suitability of the 3K strain as a probiotic candidate. Overall, the findings support the use of *E. hirae* 3K as a stable, safe, and low microbial risk probiotic strain with promising applications in sustainable aquaculture.

## Methods

### Isolation of bacterial strains

Marine sediments were collected from different locations in Alexandria and Matrouh governorates (Egypt). Bacteria were isolated using De Man Rogosa and Sharpe broth (MRS; HiMedia, India). MRS broth tubes were incubated under anaerobic conditions using the GasPak System at 37 °C for 24 h, followed by successive streaking on MRS agar plates under the same conditions. Individual phenotypically unique colonies were picked for two rounds of purification. The pure isolates were preliminarily characterized by Gram’s staining and catalase testing. The pure bacterial cultures were preserved at -20 °C in MRS broth supplemented with 50% (v/v) glycerol for further investigation.

### Morphological and biochemical characterizations

Among ten isolates (Supplementary Table S1), the 3K isolate was selected based on its superior exopolysaccharide production capacity and preliminary safety characteristics. The morphology of the 3K isolate was examined using scanning electron microscopy (SEM; JSM-IT 200) at the EM Unit, Alexandria University, Egypt. The hemolytic activity of the selected isolate was assessed by streaking on a 7% (v/v) blood agar plate, followed by incubation at 37 °C for 24 h. Following the incubation period, the plate was examined for signs of blood hemolysis. Moreover, strain 3K isolate was biochemically identified using the VITEK 2 automated microbial identification system (BioMérieux, France; http://www.biomerieux.com).

### Antimicrobial Susceptibility Testing (AST)

The antibiotic susceptibility of strain 3K was evaluated using both the minimum inhibitory concentration (MIC) method and disc diffusion assay. The test was performed using the gram-positive susceptibility card AST-P592 (BioMérieux, France) on the VITEK 2 system, following the manufacturer’s protocol. MIC values were interpreted in accordance with Clinical and Laboratory Standards Institute (CLSI) guidelines. The disc diffusion assay was carried out on Muller-Hinton agar (MHA; HiMedia, India) plates containing antibiotic discs of teicoplanin (30 µg), vancomycin (30 µg), rifampicin (5 µg), azithromycin (15 µg), cefoxitin (30 µg), linezolid (30 µg), erythromycin (15 µg), sulfamethoxazole/trimethoprim (25 µg), ceftriaxone (30 µg), colistin sulfate (10 µg), and ampicillin (10 µg) (Oxoid, UK). The plates were incubated anaerobically at 37 °C for 24 h, and inhibition zone diameters were measured in millimeters (mm). The isolates were classified as susceptible (S), intermediate (I), or resistant (R) based on CLSI breakpoints [13].

### Resistance to low pH

To evaluate acid tolerance under simulated gastric conditions, isolate 3K was cultured across different pH values. Briefly, a 1.0% (v/v) overnight culture of isolate 3K was inoculated into 20 mL of sterile MRS broth adjusted to pH 2.0, 4.0, and 6.5. Cultures were incubated at 37 °C, and growth was monitored spectrophotometrically by measuring optical density at 600 nm at hourly intervals for up to 24 h, using uninoculated MRS broth as a blank. All assays were performed in triplicate.

### Bile salt tolerance

To assess bile salt tolerance, isolate 3K was exposed to concentrations reflecting intestinal conditions. A 1.0% (v/v) overnight culture of 3K was inoculated into 20 mL of sterile MRS broth supplemented with 0.1% and 0.3% w/v of bile salts (Pallav, India). The mixture was then incubated at 37 °C for 24 h. The culture’s growth was measured at regular hourly intervals at 600 nm, with uninoculated MRS broth as a blank. This experiment was performed in triplicate.

### DNA extraction, Whole Genome Sequencing (WGS), and assembly

Genomic DNA was extracted using the HiPurA™ Bacterial Genomic DNA Purification Kit (HiMedia, India). Isolate 3K was cultivated in MRS broth and cultured at 37 °C for approximately 18 h. The isolation of genomic DNA was performed according to the manufacturer’s instructions, eluted in 10 mM Tris-HCl (pH 8.0), and the high-quality purified DNA (OD_260_/_280_ = 1.8–2.0) was used. Whole genome sequencing (WGS) was performed by MicrobesNG (Birmingham, UK; http://microbesng.uk) on an Illumina NovaSeq 6000 platform (Illumina, USA) using a 250 bp paired-end protocol with 60X sequence coverage. Raw reads were adapter-trimmed using Trimmomatic version 0.30 with a sliding window quality cut-off of Q15 [14]. *De novo* assembly is performed on samples using SPAdes version 3.7 [15]. The quality of the assembled genome sequence was assessed using the Quality Assessment Tool for Genome Assemblies (QUAST) [16].

### Genome annotation

The assembled contigs were annotated [17] using Prokka software (version 1.11). Bacterial and viral bioinformatics resource center (BV-BRC; version 3.39.3; https://www.bv-brc.org/) was used to identify protein-encoding regions and assigned functions to the genes, rRNA, tRNA, and subsystems in the genome sequence [18]. Metabolic pathways were reconstructed based on annotations from the Kyoto Encyclopedia of Genes and Genomes (KEGG; http://www.genome.jp/kegg) [19]. Clusters of Orthologous Groups (COG) functional categories were predicted (http://eggnog-mapper.embl.de) using the egg-NOG Mapper tool (version 5.0). The phylogenetic tree was created and viewed using the Bacterial Genome Tree available through the BV-BRC platform. A circular genomic map was generated with the CGView server (https://proksee.ca) to visualize the genome and annotated features [20].

### Plasmids, antibiotic resistance, and virulence genes prediction

Plasmid replicons were detected in the 3K genome sequence by the PlasmidFinder (version 2.0.1) online tool provided by the Center for Genomic Epidemiology [21]. Putative antibiotic resistance genes (ARG) were predicted using ResFinder-4.6.0 [22]. Virulence determinants were screened using the VFanalyzer platform (https://www.mgc.ac.cn), accessible through the Virulence Factor Database (VFDB).

### Prediction of bacteriocins, secondary metabolites, Exopolysaccharide (EPS) biosynthesis genes, and Carbohydrate-Active Enzymes (CAZymes)

Biosynthetic gene clusters encoding antimicrobial peptides were identified and mapped using BAGEL4 (http://bagel4.molgenrug.nl) [23]. Putative bacteriocin domains were confirmed via protein Basic Local Alignment Search Tool (BLASTp) analysis against the non-redundant protein sequence database. Furthermore, the secondary metabolite biosynthetic potential was further assessed using the antiSMASH platform (version 7.1.0) [24]. Exopolysaccharide biosynthesis gene clusters were annotated by BLASTp homology. CAZyme-associated genes in the 3K genome sequence were predicted using the dbCAN2 online tool, using a DIAMOND blast search in the Carbohydrate-Active Enzymes database (CAZy; http://www.cazy.org).

### Comparative genomic analysis

For comparative genomics, WGS of four *E. hirae* strains, namely GE5, GE2, S38-2, and F105, were retrieved from the National Biotechnology Information Center (NCBI) database (Table 1). Orthologous relationships and shared/unique gene families were analyzed using OrthoVenn3 (https://orthovenn3.bioinfotoolkits.net) [25].

**Table 1:** Bacterial strains used in this study

Strain	Accession number*	Source of isolation	Country	Collection year	Genome size (bp)	tRNA	rRNA	GC (%)
3K	JBRUYU000000000	Marine sediment	Egypt	2024	3,049,853	63	8	36.65
GE5	JADPVY000000000	Marine sediment	Italy	2018	2,753,207	61	9	36.88
GE2	JADPVX000000000	Zooplankton	Italy	2018	2,860,652	62	12	37.87
F105	JAGMUA000000000	River water	Switzerland	2021	2,765,520	62	6	36.84
S38-2	JARPYM000000000	River water	China	2022	2,816,662	61	3	36.91

*All accession numbers are from GenBank

### Antimicrobial activity

The antimicrobial activity of the cell-free supernatant (CFS) from strain 3K was assessed in vitro using the agar well diffusion assay against the indicator pathogens *Staphylococcus aureus* (ATCC 25923), *Escherichia coli* O157 (ATCC 700728), *Salmonella Typhimurium* (ATCC 14028), *Klebsiella pneumoniae* (ATCC 33495), and *Pseudomonas aeruginosa* (ATCC 27853), following established protocols [11, 26]. CFS was prepared by centrifuging a stationary-phase culture at 8,000 rpm for 10 min at 4 °C. Then, the supernatant was collected, adjusted to pH 7.0, heated at 90 °C for 2 min, and sterilized by filtration through a 0.45 μm membrane filter. Overnight cultures of each pathogen were uniformly spread onto Mueller-Hinton agar (MHA) plates, wells were aseptically created, and 100 µL of prepared CFS was added per well. Chloramphenicol (100 µg/mL) and sterile MRS broth served as positive and negative controls, respectively. Plates were incubated at 37 °C for 24 h, after which inhibition zones were measured in mm [27].

For morphological analysis, pathogens were exposed to sterile CFS for 12 h. Cells were fixed with 5% glutaraldehyde, subjected to gold sputter-coating (15 Å for 2 min), and visualized by scanning electron microscopy (SEM; JSM-IT 200, JEOL, Japan) at an accelerating voltage of 20.0 kV [28, 29].

### Statistical analysis

All experiments were conducted in triplicate, and results are presented as the mean ± standard deviation (SD). Data were processed using Microsoft Excel, and statistical significance was evaluated by one-way analysis of variance (ANOVA). Differences were considered statistically significant at *p* < 0.05.

## Results

### Morphological, biochemical identification, and hemolytic activity of isolate 3K

**Fig. 1:**
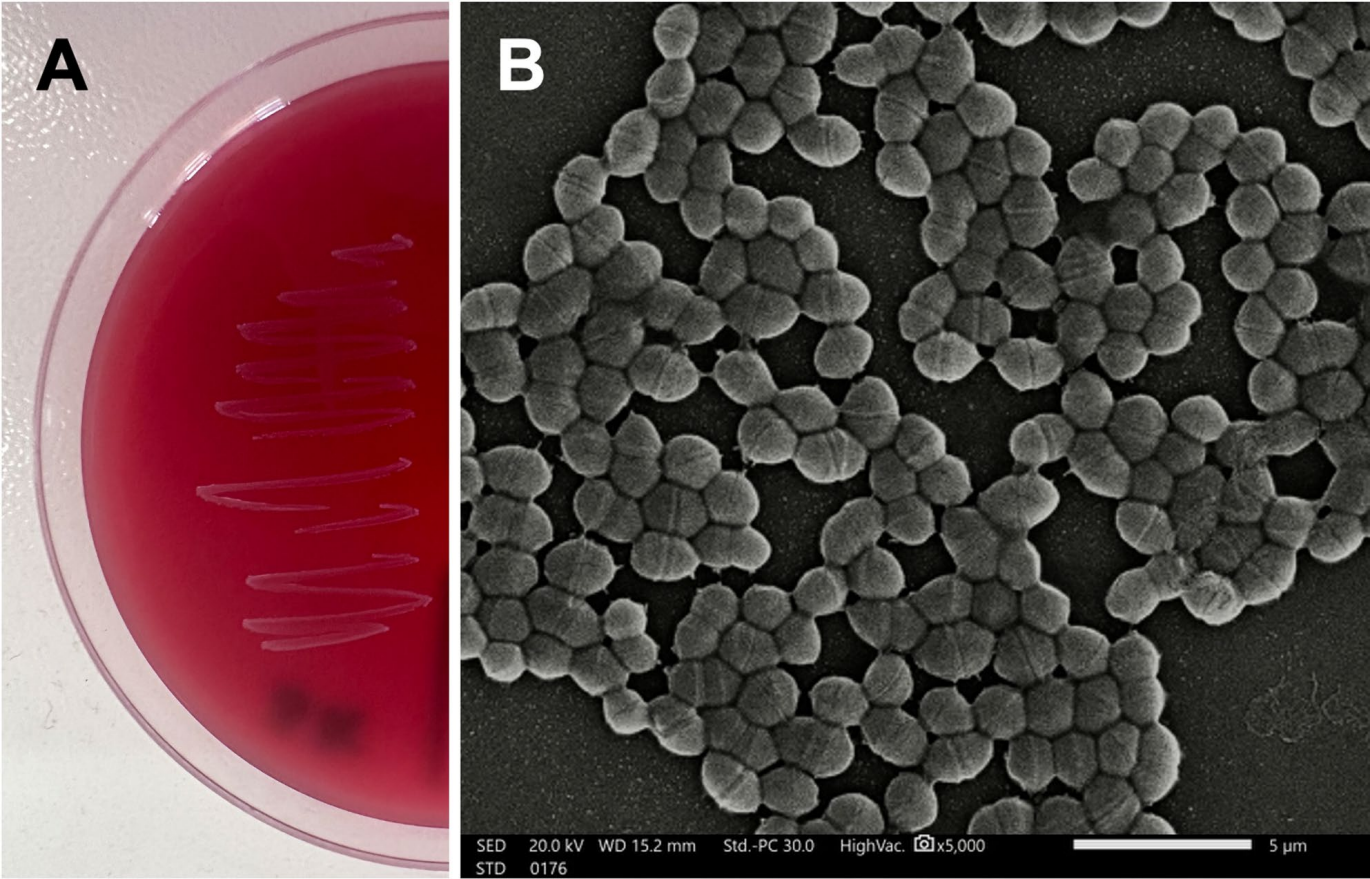
**A** Growth of 3K strain on the surface of blood agar following 24 h of incubation at 37 °C, **B** Scanning electron micrograph illustrating the coccoidal cell morphology of isolate 3K as observed by scanning electron microscope

Isolate 3K was characterized as gamma-hemolytic, showing no hemolytic activity on blood agar (Fig. 1A). Coccoidal-shaped Isolate 3K was found to be gram-positive in response to Gram stain (Fig. 1B). Subsequent identification using the VITEK 2 revealed the isolate was found to be *Enterococcus* sp., with a 97% similarity probability. Furthermore, biochemical characterization of the 3K isolate revealed several metabolic functions, as shown in Table 2. The isolate exhibited positive responses towards several carbohydrate fermentation tests, including D-galactose, D-ribose, N-acetyl-D-glucosamine, D-maltose, D-mannose, D-raffinose, sucrose, and D-trehalose. In contrast, the strain was negative for most glycosidase and arylamide activities, such as β-galactosidase, α-glucosidase, and leucine arylamides, as well as for urease and phosphatase, indicating limited hydrolytic enzyme activity. Furthermore, the strain showed tolerance to high concentrations of NaCl (6.5%), underlining its adaptability to the marine environment.

**Table 2:** Biochemical characterization of 3K marine isolate using VITEK 2 system

Test	Result	Test	Result
D-AMYGDALIN (AMY)	-	UREASE (URE)	-
PHOSPHATIDYLINOSITOL PHOSPHOLIPASE C (PIPLC)	-	D-GALACTOSE (dGAL)	+
D-XYLOSE (dXYL)	-	D-RIBOSE (dRIB)	+
ARGININE DIHYDROLASE 1 (ADH1)	+	L-LACTATE alkalinization (ILATK)	-
BETA-GALACTOSIDASE (BGAL)	-	LACTOSE (LAC)	-
ALPHA-GLUCOSIDASE (AGLU)	-	N-ACETYL-D-GLUCOSAMINE (NAG)	+
Ala-Phe-Pro ARYLAMIDASE (APPA)	-	D-MALTOSE (dMAL)	+
CYCLODEXTRIN (CDEX)	-	BACITRACIN RESISTANCE (BACI)	-
L-Aspartate ARYLAMIDASE (AspA)	-	NOVOBIOCIN RESISTANCE (NOVO)	+
BETA GALACTOPYRANOSIDASE (BGAR)	-	GROWTH IN 6.5% NaCl (NC6.5)	+
ALPHA-MANNOSIDASE (AMAN)	-	D-MANNITOL (dMAN)	-
PHOSPHATASE (PHOS)	-	D-MANNOSE (dMNE)	+
Leucine ARYLAMIDASE (LeuA)	-	METHYL-B-D-GLUCOPYRANOSIDE (MBdG)	-
L-Proline ARYLAMIDASE (ProA)	-	PULLULAN (PUL)	-
BETA GLUCURONIDASE (BGURr)	-	D-RAFFINOSE (dRAF)	+
ALPHA-GALACTOSIDASE (AGAL)	-	O/129 RESISTANCE (comp. vibrio.) (O129R)	+
L-Pyrrolydonyl-ARYLAMIDASE (PyrA)	-	SALICIN (SAL)	+
BETA-GLUCURONIDASE (BGUR)	-	SACCHAROSE/SUCROSE (SAC)	+
Alanine ARYLAMIDASE (AlaA)	-	D-TREHALOSE (dTRE)	+
Tyrosine ARYLAMIDASE (TyrA)	-	ARGININE DIHYDROLASE 2 (ADH2s)	+
D-SORBITOL (dSOR)	-	OPTOCHIN RESISTANCE (OPTO)	+
POLYMIXIN B RESISTANCE (POLYB)	+		

(+) Positive, (−) Negative

### Antimicrobial Susceptibility Test (AST)

The antibiotic susceptibility profile of isolate 3K was determined using the disc diffusion method (Supplementary Table S2), and minimum inhibitory concentration (MIC) values for clinically relevant antibiotics were assessed via VITEK 2 analysis (Table 3). The strain exhibited broad-spectrum susceptibility across multiple antibiotic classes. The strain was susceptible to ampicillin with an MIC value of ≤ 2 µg/mL. Among fluoroquinolones, ciprofloxacin demonstrated an MIC value of ≤ 0.5 µg/mL. Moreover, macrolide testing revealed susceptibility to erythromycin (0.5 µg/mL). Furthermore, the isolate was sensitive to oxazolidinone (linezolid, 2 µg/mL), glycopeptides (teicoplanin and vancomycin, ≤ 0.5 µg/mL), tetracycline (≤ 1 µg/mL), and tigecycline (≤ 0.12 µg/mL).

**Table 3:** Minimum Inhibitory Concentration (MIC) interpretation of *Enterococcus hirae* 3K detected by VITEK 2 as per Clinical and Laboratory Standards Institute (CLSI) guidelines

Antibiotic	Interpretation*of MIC value
Ampicillin	≤ 2 (S)
Gentamicin High Level (Synergy)	SYN-S (S)
Streptomycin High Level (Synergy)	SYN-S (S)
Ciprofloxacin	≤ 0.5 (S)
Erythromycin	0.5 (S)
Linezolid	2 (S)
Teicoplanin	≤ 0.5 (S)
Vancomycin	≤ 0.5 (S)
Tetracycline	≤ 1 (S)
Tigecycline	≤ 0.12 (S)

*Interpretation of MIC values. (S) Susceptible

### Bile and acid tolerance

**Fig. 2:**
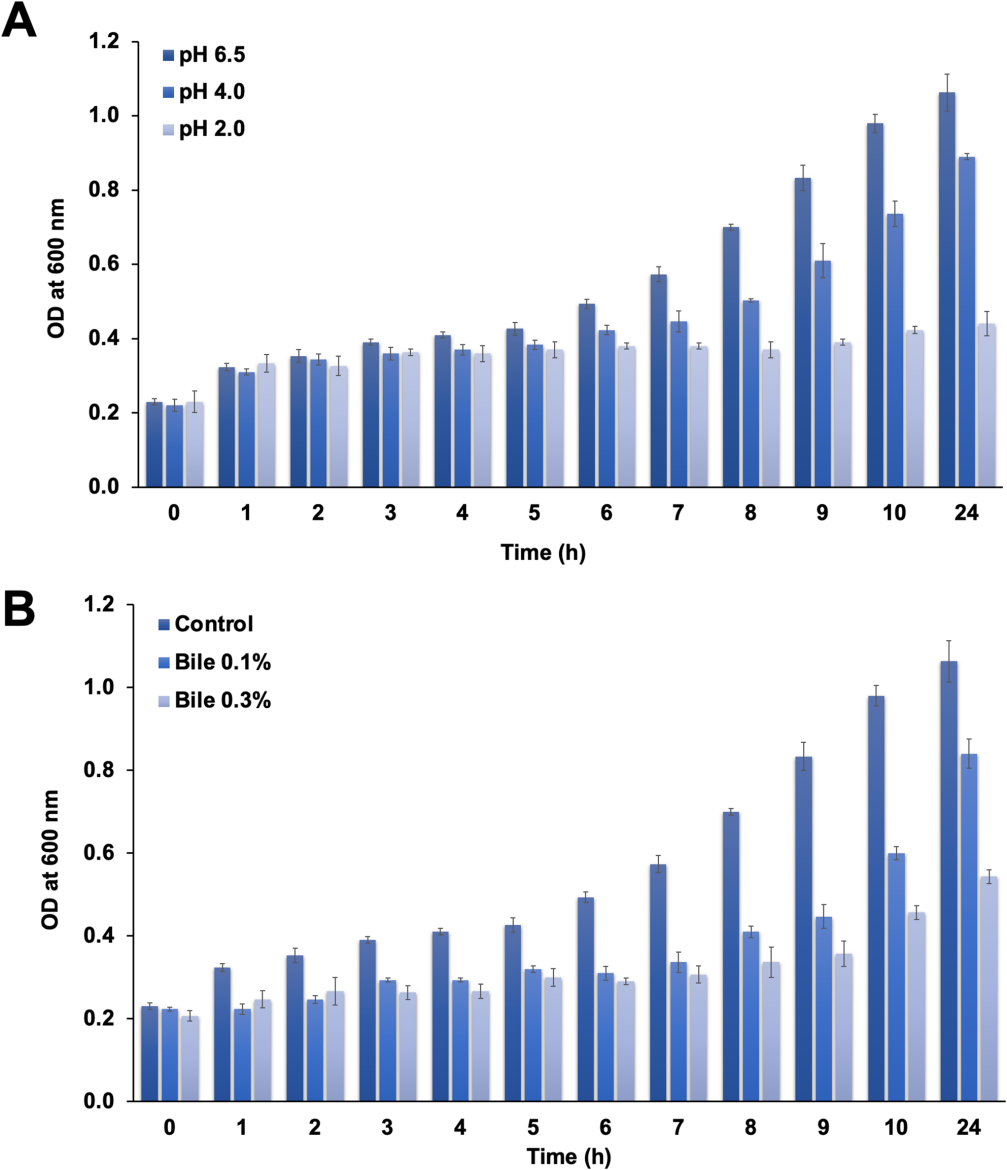
**A** Survival of isolate 3K under different pH conditions (2.0, 4.0, and 6.5), **B** Growth in MRS broth supplemented with varying concentrations of bile salts (0.1 and 0.3%) over different incubation time intervals at 37 °C. The growth was monitored hourly by measuring the optical density at 600 nm. Results are expressed as mean ± standard deviation of three replicates

Strain 3K was evaluated for tolerance to simulated gastrointestinal stress conditions. The isolate demonstrated significant survival under acidic conditions, maintaining viability at low pH values over time (Fig. 2A). Furthermore, it exhibited sustained growth in the presence of 0.1% and 0.3% (w/v) bile salts across a 24 h (Fig. 2B). These results indicate that strain 3K possesses robust physiological resilience, enabling it to withstand harsh environmental stresses relevant to gastrointestinal tract.

### General genomic features

**Fig. 3:**
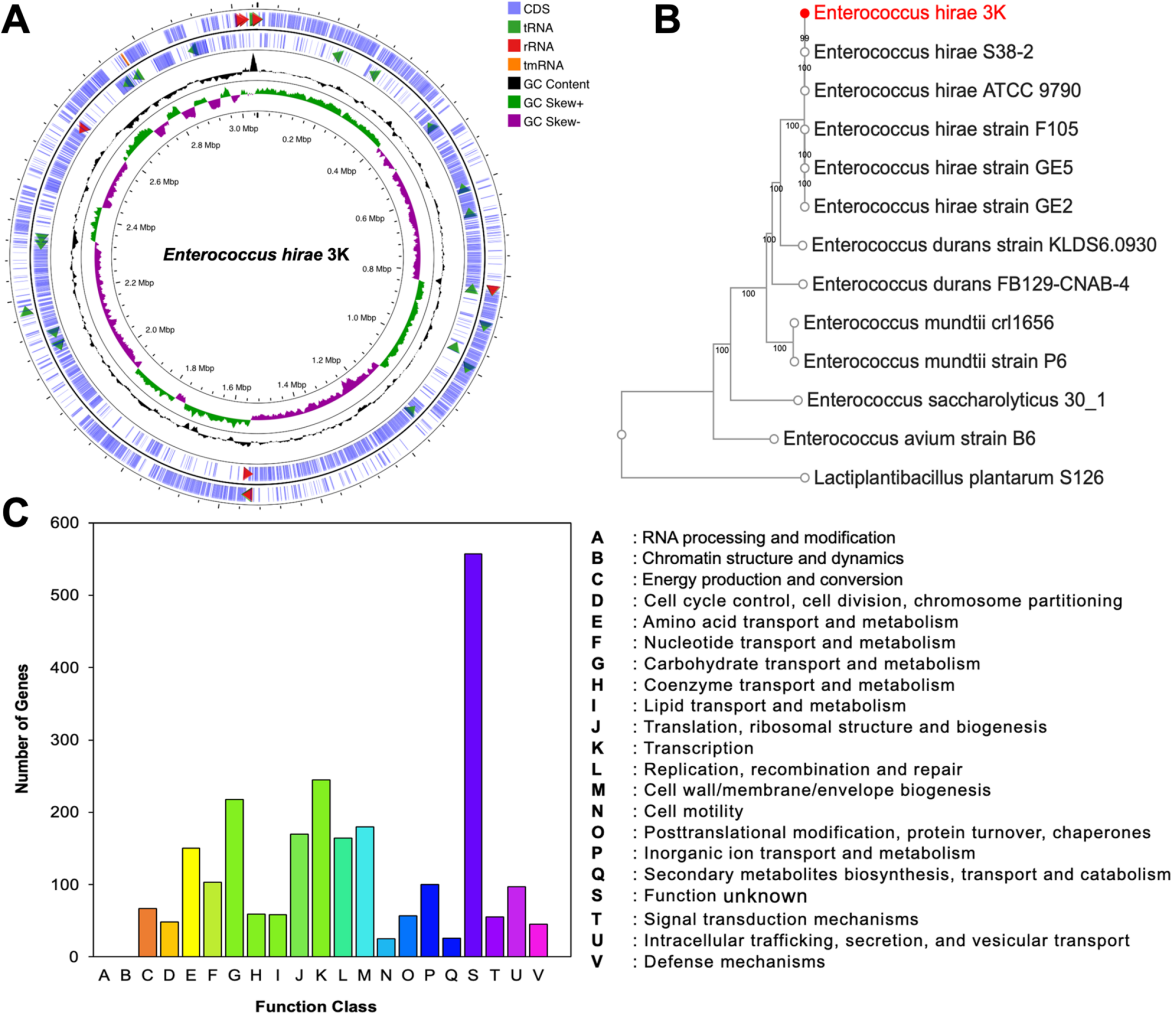
**A** Circular draft genomic map illustrating the contig sequences of *Enterococcus hirae* 3K, **B** Whole genome-based phylogenetic tree showing the relationship of strain 3K to its closest related strains retrieved from the NCBI database, **C** Functional classification of predicted proteins of strain 3K based on cluster of orthologous groups (COG)

The whole-genome sequencing of the 3K strain generated 1,992,994 reads with a median insert size of 627 bases. A total genome length of approximately 2.91 Mb, assembled into 89 contigs, of which 34 contigs were ≥ 1 kb. Assembly contiguity was supported by an N50 value of 213,022 bp and an L50 of 5, with the largest contig measuring 504,531 bp. Moreover, predicted 2,885 protein-coding sequences (CDS) and a G + C content of 36.65% were estimated in the genome. Using the BV-BRC platform, genomic features, including 63 tRNA genes and 8 rRNA genes, were predicted in the 3K genome sequence. Moreover, the COG functional annotation identified genes distributed across 23 functional categories. The KEGG pathways analysis revealed abundant functional categories of genes associated with carbohydrate metabolism. Otherwise, the genome included pathways related to lipids and xenobiotic metabolic pathways. The phylogenetic analysis revealed that the 3K strain is closely related to *Enterococcus hirae* strains (Fig. 3). Otherwise, based on the whole genome-based phylogenetic tree, marine isolate 3K revealed a high degree of genomic similarity with bootstrap values of 99–100%. *E. hirae* 3K shares a close evolutionary lineage with S38-2 (accession no. *JARPYM000000000*), F105 (accession no. *JAGMUA000000000*), GE2 (accession no. *JADPVX000000000*), GE5 (accession no. *JADPVY000000000*), and ATCC 9790.

### Assessment of strain 3K genome safety

Genetic determinants associated with safety were investigated in the draft genome of *E. hirae* 3K strain. Analysis revealed the absence of key virulence determinants, including genes encoding cytolysin (*cylA*), hyaluronidase (*hyl*), gelatinase (*gelE*), aggregation (*agg*), and adhesion collagen protein (*ace*). Additionally, screening for antibiotic resistance genes identified no clinically critical determinants, such as vancomycin-resistance genes (*vanA*, *vanB*,* vanC*). However, one intrinsic aminoglycoside resistance gene, namely, *aac*(6’)-Iid, was predicted in the 3K genome sequence. Moreover, two plasmids’ replicons were predicted, namely rep18a (accession no. *AB158402*) and rep11a (accession no. *AB178871*).

### Determination of probiotic-associated genes

Genomic annotation of *E. hirae* 3K revealed a complement of genes linked to probiotic functionality. The genome contained the bile tolerance-related genes cholylglycine hydrolase (*cbh*) and cyclopropane-fatty-acyl-phospholipid synthase (*cfa*). Genes involved in adhesion and biofilm formation, including sortase A (*srtA*) and enolase (*eno*), were also detected. Furthermore, several genes related to acid tolerance were present, such as the gamma-aminobutyrate antiporter (*gadC*), Na⁺/H⁺ antiporters (*nhaC*,* napA*), acid-resistant locus arl7 (*yeiC*), and the H⁺/Cl⁻ exchange transporter (*clcA*). The genome also encoded a complete set of stress response genes, including the heat-inducible transcriptional repressor (*hrcA*), molecular chaperones (*dnaK*, *dnaJ*), and heat shock proteins (*grpE*, *groES*, *groEL*). The genome also encoded pathways for the biosynthesis of essential vitamins, such as dihydrofolate reductase (*folA*) for folate production, riboflavin synthase (*ribE*) for riboflavin synthesis, biotin synthase (*bioB*) for biotin formation, and thiamine-phosphate synthase (*thiE*) for thiamine biosynthesis. Antioxidant-related genes, such as superoxide dismutase *(sodA*), glutathione reductase (*gor*), and thioredoxin (*trx*), were also identified in the 3K genome sequence.

### Detection of bacteriocins and secondary metabolites genes

**Fig. 4:**
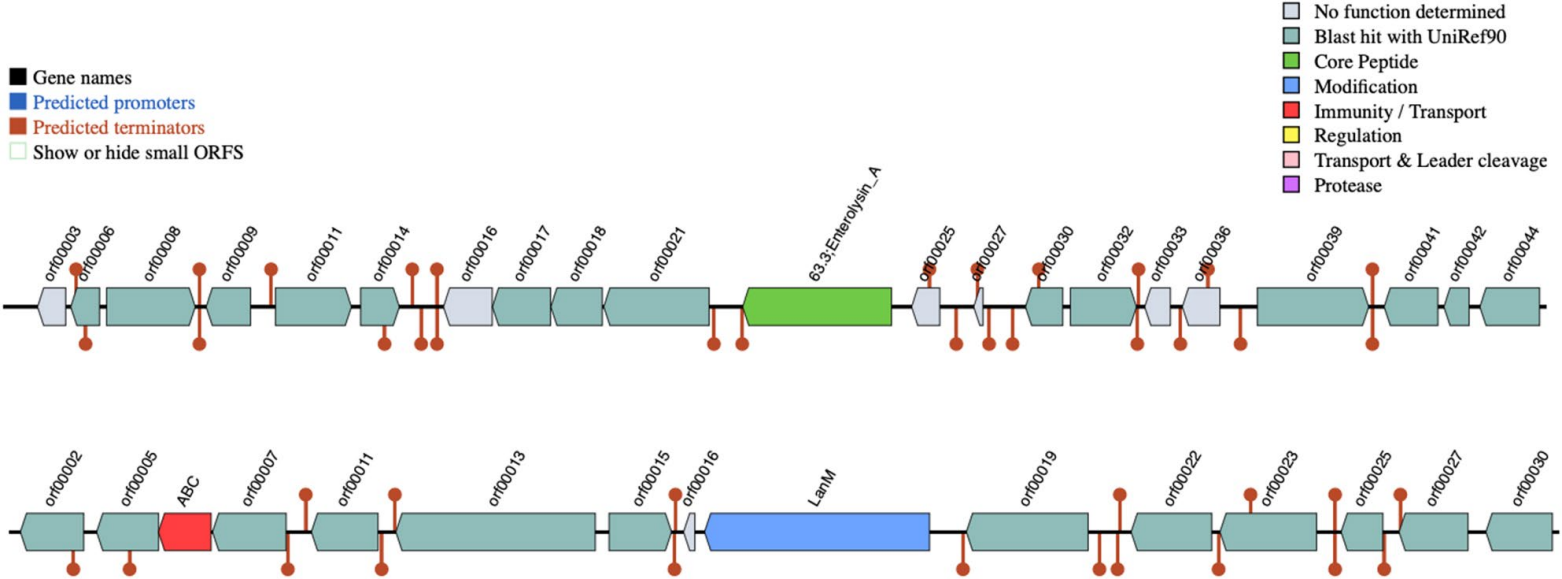
Putative bacteriocin-associated gene clusters, including Enterolysin A (EnlA) and Lanthipeptide (LanM), were predicted using the BAGEL4 webserver

**Fig. 5:**
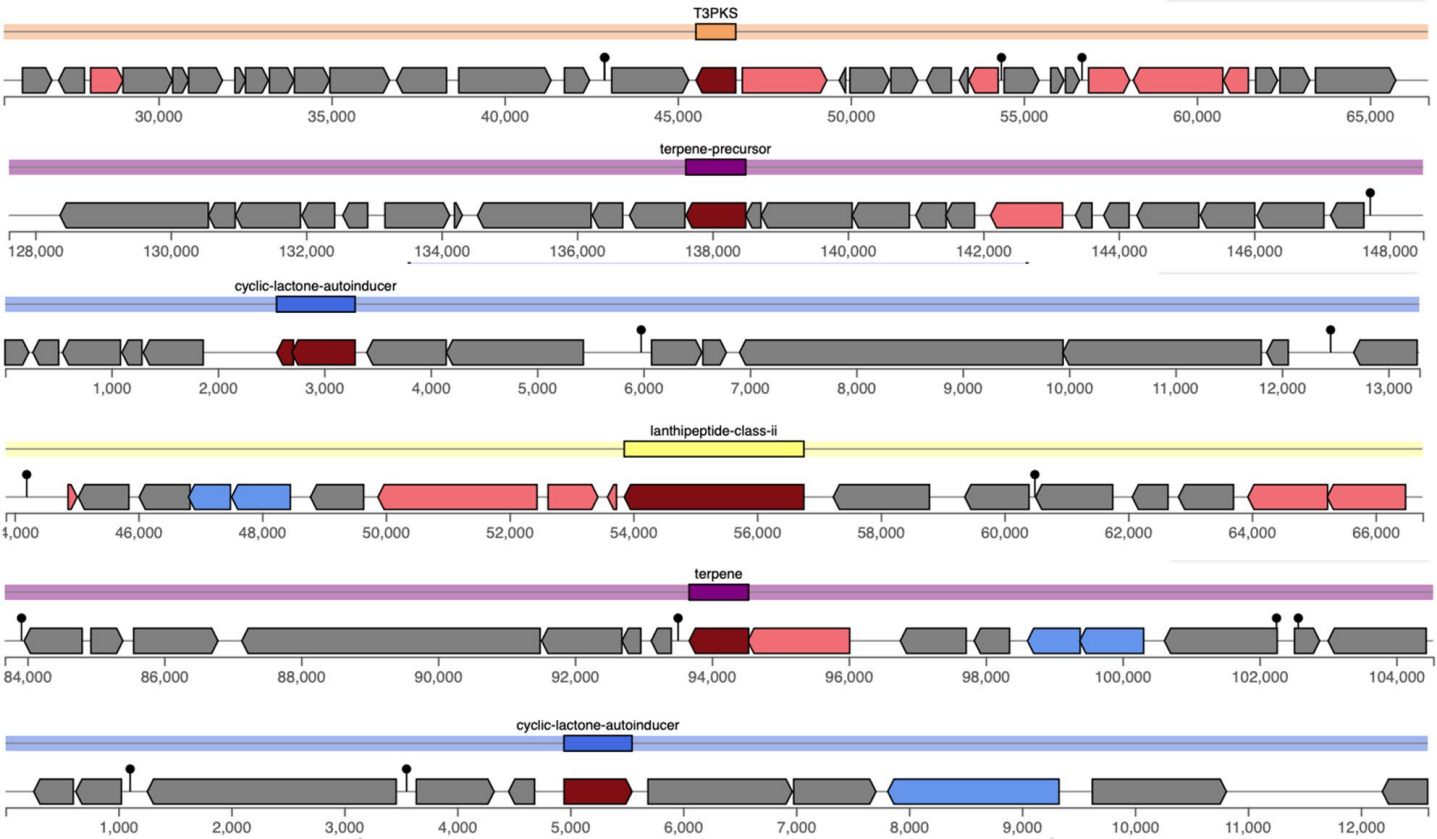
Prediction of secondary metabolite biosynthetic gene clusters identified by antiSMASH platform

In silico analysis using BAGEL4 identified genetic loci encoding enterolysin A class III (EnlA) and lanthipeptide class II (LanM) bacteriocins in 3K genome sequence (Fig. 4). The genome analysis revealed the presence of multiple biosynthetic gene clusters potentially involved in secondary metabolite production (Fig. 5). The predicted clusters include a type III polyketide synthase (T3PKS), terpene and terpene precursor pathways, a lanthipeptide (class II) cluster, as well as cyclic lactone autoinducer systems. The T3PKS cluster contained several core biosynthetic genes. Similarly, the terpene clusters displayed characteristic genes responsible for isoprenoid backbone formation and modification.

### Prediction of EPS biosynthesis-related genes based on genome analysis

Genome analysis of *E. hirae* 3K revealed two distinct gene clusters associated with exopolysaccharide (EPS) and capsular polysaccharide biosynthesis. The first cluster comprised nine genes, including *ywqCDE*, encoding a probable capsular polysaccharide biosynthesis protein, a tyrosine-protein kinase, and a tyrosine-protein phosphatase, respectively, together with *pglF* (UDP-*N*-acetyl-α-D-glucosamine dehydratase), *pglC* (undecaprenyl phosphate transferase), *epsM* (acetyltransferase), *epsN* (aminotransferase), *epsD* (glycosyltransferase), and *ugd* (UDP-glucose 6-dehydrogenase), which are involved in sugar precursor biosynthesis and polysaccharide modification. A second EPS-related cluster was also identified, including *ywqCDE*, *wcaJ* (UDP-glucose transferase), *epsF* and *epsJ* (putative glycosyltransferases), *tll* (dTDP-6-deoxy-L-talose 4-dehydrogenase), *ugd* (UDP-glucose 6-dehydrogenase), and *murJ* (lipid II flippase). Collectively, these clusters encode enzymes responsible for EPS synthesis, structural modification, and membrane translocation.

### Identification of active carbohydrate enzymes

To characterize the carbohydrate metabolic potential of *E. hirae* 3K, its genome was annotated using the CAZy database. The CAZyme profile revealed the distribution of enzymatic classes involved in carbohydrate processing (Supplementary Fig. 1). The largest proportions are represented by glycosyltransferases (GTs) and glycoside hydrolases (GHs), each comprising 40.26% of the total CAZyme content. Carbohydrate-binding modules (CBMs) account for 10.39%. Auxiliary activities (AAs) constitute 5.19%. Carbohydrate esterases (CEs) represent the smallest group, at 2.60%.

### Comparative genome analyses

**Fig. 6:**
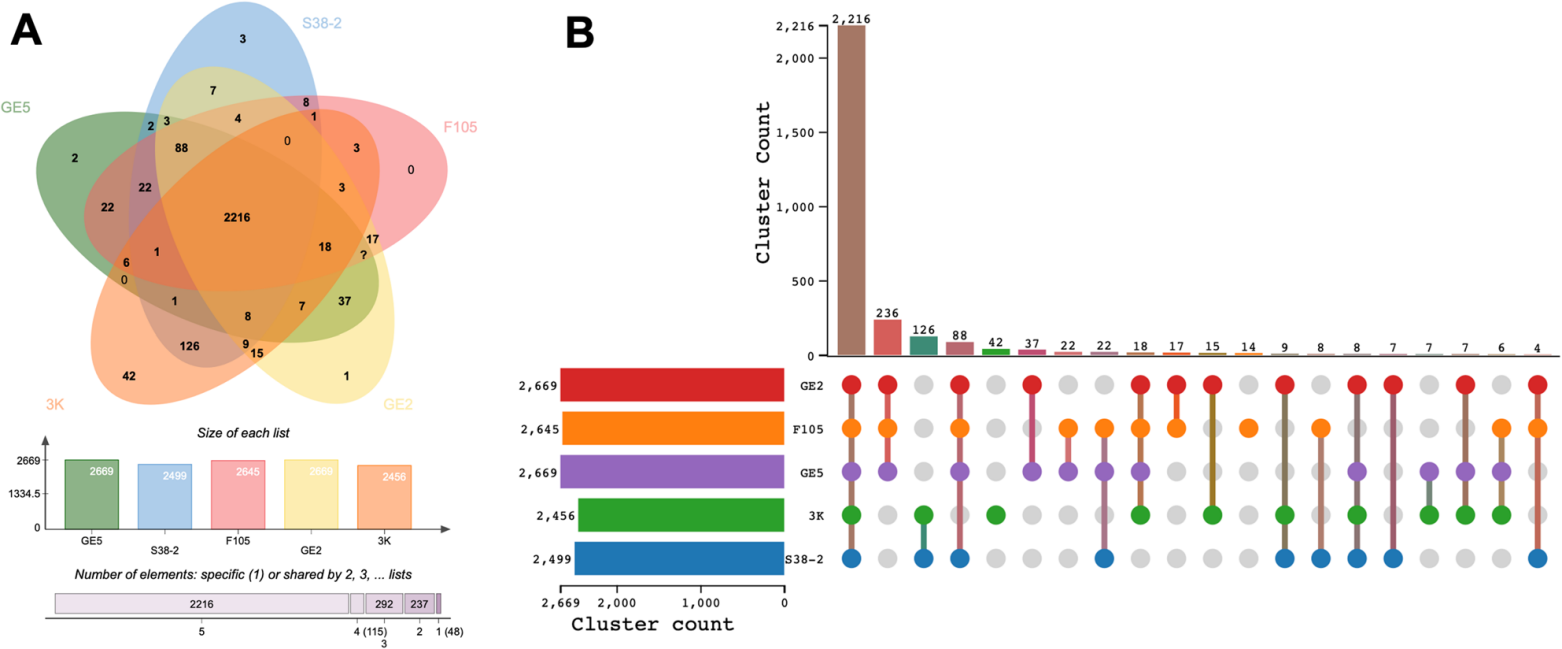
**A** Venn diagram illustrating shared and unique gene clusters among *Enterococcus hirae* 3K and other genomes (GE5, S38-2, F105, and GE2). **B** Upset plot showing the distribution of unique genes across each strain

A comparative genome analysis was conducted between the 3K isolate and four other *E. hirae* strains (GE5, S38-2, F105, and GE2) derived from distinct sources (Fig. 6). An UpSet plot, representing a quantitative assessment of shared gene content, identified 2,216 orthologous gene clusters common to all analyzed *E. hirae* strains. Strain-specific clusters were also identified, with the largest numbers of unique genes present in strains GE2 and F105.

### Antimicrobial activity

**Fig. 7:**
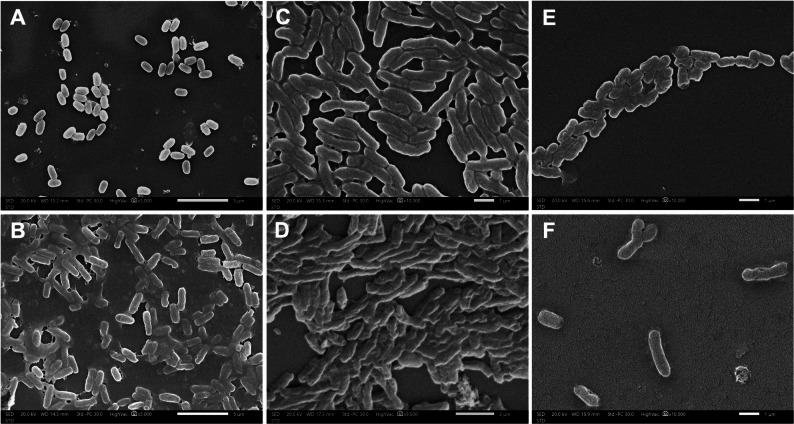
Micrographs obtained using scanning electron microscopy (SEM) showing the morphology of untreated control bacteria (**A**,** C**,** E**) and treated bacteria with the cell-free supernatant (CFS) of strain 3K for 12 h (**B**,** D**,** F**). Panels (**A**,** B**) represent *Klebsiella pneumoniae*, (**C**,** D**) represent *Salmonella Typhimurium*, (**E**,** F**) represent *Pseudomonas aeruginosa*

The sterile CFS of *E. hirae* 3K exhibited potent antimicrobial activity against the gram-negative pathogens *K. pneumoniae*, *P. aeruginosa*, and *S. Typhimurium*, but no inhibitory effect was observed against *S. aureus* and *E. coli* O157. Comparative scanning electron microscopy of control versus 3K CFS-treated cells confirmed a bacteriolytic mode of action (Fig. 7). Untreated controls displayed intact, morphologically typical cells. In contrast, CFS exposure induced extensive cellular damage across all three pathogens. *K. pneumoniae* cells exhibited membrane rupture, cytoplasmic leakage, shrinkage, and debris formation. *P. aeruginosa* cells showed pronounced membrane disruption, culminating in complete lysis and a marked reduction in cell density. Similarly, *S. Typhimurium* cells displayed substantial surface damage and structural degradation.

## Discussion

Probiotics have gained considerable attention for their crucial roles in the development of current therapeutics and value-added, newly discovered natural antimicrobials [30]. Marine-derived probiotics exhibit remarkable adaptability to extreme environmental conditions, enabling them to develop unique physiological features and synthesize distinctive bioactive metabolites with notable biological activities and potential industrial applications, particularly in the food industry and in aquaculture [31]. Furthermore, comprehensive genomic analysis of the novel marine probiotic strains is strongly recommended, as it provides deep insights into their biotechnological usefulness, functional diversity, metabolic pathways control, and other health-promoting mechanisms [32]. Consequently, this study employed a genome-based approach to evaluate the safety profile and probiotic potential of *Enterococcus hirae* 3K, a marine sediment-derived isolate from Egypt.

The morphological and biochemical characterization, along with the genomic profile, strongly supports classification of strain 3K as a marine probiotic with a favorable safety profile. The absence of hemolytic activity indicates a non-pathogenic nature and aligns with the gamma-hemolytic profile commonly associated with non-virulent *Enterococcus* strains. The findings agree with a previous study of Adnan et al. [11], which demonstrated the non-hemolytic potential of probiotic strain *E. hirae* F2, isolated from the gut of freshwater fish. Moreover, *E. hirae* 3K reveals tolerance to salt, reflecting its adaptation to the marine environmental conditions, which is advantageous for potential applications in the aquaculture ecosystem.

Whole-genome sequencing of *E. hirae* 3K revealed genetic and functional attributes consistent with prior reports for this species [33, 34]. Functional annotation of the 3K genome identified genes encoding proteins for carbohydrate metabolism, a core set of probiotic functions strongly associated with LAB strains [6]. Furthermore, the genome was found to harbor several genes associated with probiotic mechanisms. The presence of cholylglycine hydrolase (converts conjugated bile acid into free bile acid) and cyclopropane fatty acyl phospholipid synthase genes (enhances lipid synthesis) suggests the capability of the 3K strain to survive in the presence of 0.3% bile salt [35]. Furthermore, the detection of genes associated with acid tolerance indicated the ability of 3K to survive extreme acidic conditions. The mechanism involves the manipulation of the proton transport to maintain a lower intercellular concentration of protons [36]. Moreover, adhesion and biofilm formation are essential to probiotic colonization and host interaction. Sortase enzyme promotes intestinal colonization by enterococci, while enolase acts as an adhesin in lactobacilli [37, 38]. Additionally, the genome encodes an array of stress response genes that play a role at high temperatures and reveal a defense mechanism against sudden heat shock stress [39]. The predicted biosynthetic potential for B-group vitamins, including folate, riboflavin, biotin, and thiamine, is a desirable trait of probiotics. This enhances their functional potential and provides health benefits to the host. The presence of antioxidant-related genes indicates the potential of the 3K strain to mitigate oxidative stress. Together, superoxide dismutase, glutathione reductase, and thioredoxin participate in the 3K strain’s oxidative stress tolerance [40].

The genome of enterococci contains several widespread virulence-associated genes. For instance, clinically significant isolates such as *Enterococcus faecalis* frequently harbor the virulence-associated genes such as *gelE* (encoding a gelatinase) *and esp* (encoding an enterococcal surface protein linked to biofilm formation and pathogenesis) [41]. The absence of these genes in the *E. hirae* 3K genome further supports its safety profile and underscores its potential as a probiotic candidate. Moreover, our results revealed the occurrence of aminoglycoside antibiotic-resistant gene, *aac*(6’)-Iid, in the 3K genome sequence, which is intrinsic in *E. hirae* [42]. The lack of clinically relevant vancomycin antibiotic resistance determinants (*vanA*, *vanB*,* vanC*) supports the strain’s safety and reinforces its low-risk profile for horizontal transfer [43]. These findings agree with the previous study of Li et al. [3] that reports the absence of pathogenic determinants in *E. hirae* strains. Notably, while plasmid replicons were detected in the 3K genome, none were associated with high-risk antibiotic resistance genes. This characteristic supports a stable genomic profile with a reduced likelihood of disseminating concerning genetic determinants via horizontal gene transfer (HGT). The genetic stability of a probiotic candidate constitutes a fundamental criterion in its safety evaluation [44].

The antimicrobial activity observed for the neutralized CFS of strain 3K suggests a potential bacteriocin synthesis capability. This is corroborated by the in silico identification of bacteriocin-associated biosynthetic gene clusters within the 3K genome sequence. Enterolysin A is reported to exhibit a bacteriolytic mode of action through peptidoglycan hydrolysis [45, 46], while lanthipeptides typically act via membrane disruption [47]. The co-occurrence of these genetic determinants implies a genomic basis for broad-spectrum antimicrobial potential, a feature noted in other *E. hirae* strains where such clusters may contribute to ecological fitness [41].

The genomic profile of strain 3K reveals a repertoire of biosynthetic gene clusters (BGCs), indicating a substantial potential for producing bioactive secondary metabolites. Specifically, a type III polyketide synthase (T3PKS) cluster was identified, suggesting an ability to synthesize polyketide compounds known to mediate microbial stress adaptation [48]. Furthermore, the detection of cyclic lactone autoinducer-associated BGCs implies a genetic basis for producing signaling molecules involved in quorum sensing and biofilm dynamics [49, 50]. Distinct gene clusters for exopolysaccharide (EPS) biosynthesis were also annotated, which are predicted to confer ecological advantages, including enhanced tolerance to desiccation, osmotic fluctuation, and phage predation [51]. This functional prediction is corroborated by a notable abundance of glycosyltransferase (GT) genes within the CAZyme profile, a genetic signature commonly linked to EPS polymerization and export [52]. Collectively, these genomic features, alongside a broader suite of CAZymes essential for processing complex carbohydrates, underpin a robust adaptive strategy for host persistence and environmental resilience [53].

Comparative genomic analysis between *E. hirae* 3K and four other *E. hirae* strains revealed a high level of genomic conservation, indicating the genetic stability within the species. Nonetheless, the presence of unique gene clusters in strains GE2 and F105 suggests that strain-specific genomic variations may confer distinct ecological or functional adaptations. The identification of both conserved and unique genomic elements emphasizes the genetic diversity within *E. hirae*, reflecting its ability to inhabit various ecological niches, including marine environments.

The genomic profile of *Enterococcus hirae* 3K harbors a promising repertoire of genes linked to putative probiotic functions. However, this in silico characterization lacks direct host-based validation, which is essential to substantiate its functional efficacy, colonization dynamics, and host-microbe interactions within a physiologically relevant gastrointestinal environment. Consequently, these genomic predictions require future in vivo validation in appropriate animal models to conclusively establish the strain’s safety, colonization capacity, and host-derived benefits. Furthermore, subsequent research must address regulatory frameworks for probiotic substantiation and approval, as these considerations are critical for translating genomic potential into validated commercial or therapeutic applications.

## Conclusion

This study provides insight into the genotypic patterns of safety and probiotic potential in *E. hirae* 3K, isolated from marine sediments in Egypt. This Isolate exhibited a significant antimicrobial activity against several pathogens, confirming its antimicrobial efficacy. On the other hand, the assembled genome of this strain has provided clear evidence of virulence and antibiotic resistance, consistently indicating that strain 3K is safe, stable, and poses low microbial risk, according to in-silico and phenotypic investigations. Nevertheless, in vivo studies are warranted to evaluate the probiotic efficacy in an animal model. This strain can be used as a probiotic-producing candidate for biotechnological applications and in aquaculture.

## Supplementary Information

Supplementary Material 1.

Supplementary Material 2.

## Data Availability

The dataset analyzed during the current study is available in the DDBJ/ENA/GenBank database GenBank repository, with the primary accession number **JBRUYU000000000** .

## Abbreviations

ARG: Antibiotic Resistance Genes
CDS: Coding Sequence
CFS: Cell-Free Supernatant
CLSI: Clinical and Laboratory Standards Institute
COG: Clusters of Orthologous Groups
EPS: Exopolysaccharide
KEGG: Kyoto Encyclopedia of Genes and Genomes
LAB: Lactic Acid Bacterium
MRS: De Man–Rogosa–Sharpe
PGAP: Prokaryotic Genome Annotation Pipeline
SEM: Scanning Electron Microscopy
WGS: Whole Genome Sequencing
