# Supplementary figures and images for: Genomic insights into probiotic functionality of Enterococcus hirae 3K isolated from Egyptian coastal sediments with special reference to exopolysaccharide production and antimicrobial activity potential
Source: BMC Microbiol. 2026 Mar 5;26:321. doi: 10.1186/s12866-026-04830-1 (PMC13063575; doi:10.1186/s12866-026-04830-1)

**
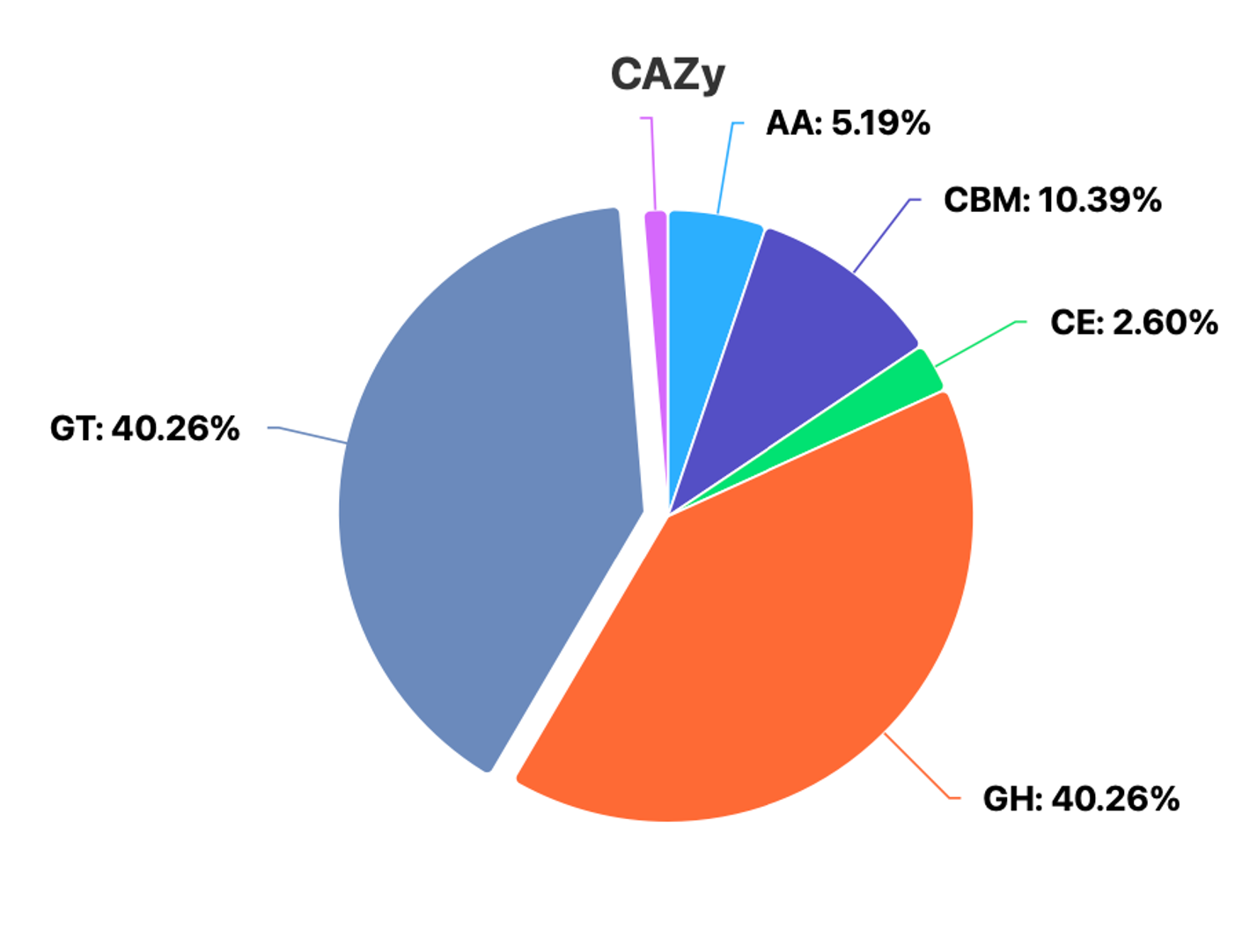
**

**Supplementary Figure 1. CAZyme distribution in the *Enterococcus hirae* 3K genome sequence.**

Supplement: Supplementary file 1 — Supplementary Material 1. [file 12866_2026_4830_MOESM1_ESM.docx]
