# Supplementary material for: Genomic insights into probiotic functionality of Enterococcus hirae 3K isolated from Egyptian coastal sediments with special reference to exopolysaccharide production and antimicrobial activity potential
Source: BMC Microbiol. 2026 Mar 5;26:321. doi: 10.1186/s12866-026-04830-1 (PMC13063575; doi:10.1186/s12866-026-04830-1)
Supplement: Supplementary file 2 — Supplementary Material 2. [file 12866_2026_4830_MOESM2_ESM.docx]

| **Sample code** | **Source of isolation** | **Growing on MRS medium** | **Catalase reaction** | **Morphology, Gram staining reaction** | **EPS production ^c^** |
| --- | --- | --- | --- | --- | --- |
| **1** | El- Shatby ^a^ | + | - | Cocci (+ve) | **++** |
| **2** | El- Shatby ^a^ | - | non | non | **-** |
| **3K** | Matrouh ^b^ | + | - | Cocci (+ve) | **++++** |
| **4** | Bahary ^a^ | - | non | non | **-** |
| **5** | Gleem ^a^ | + | + | non | **++** |
| **6** | El- Shatby ^a^ | - | non | non | **-** |
| **7** | Gleem ^a^ | - | non | non | **-** |
| **8** | El- Shatby ^a^ | - | non | non | **-** |
| **9** | Bahary ^a^ | + | - | Bacilli (+ve) | **+++** |
| **10** | Matrouh ^b^ | + | - | Cocci (+ve) | **++** |

**Supplementary Table S1**. Phenotypic Characteristics of Potential Probiotic Strains Isolated from Marine Sediments.

^a^ El-Shatby, Gleem, Bahary, Alexandria governorate.

^b^ Matrouh, Matrouh governorate.

^c^ MRS media broth was used for the growth and production of EPS at 37°C.

**Supplementary Table S2.** Antibiotic sensitivity of 3K isolate to selected antibiotics using disc diffusion method**.**

|  | **Diameter of inhibition zone (mm)/Antibiotic resistance*** |
| --- | --- |
| Teicoplanin (TEI, 30) | **S** |
| Vancomycin (VA, 30) | **S** |
| Rifampicin (RD, 5) | **S** |
| Azithromycin (AZM, 15) | **S** |
| Cefoxitin (FOX, 30) | **R** |
| Linezolid (LZ, 30) | **S** |
| Erythromycin (E, 15) | **S** |
| Sulfamethoxazole/Trimethoprim (SXT, 25) | **R** |
| Ceftriaxone (CTR, 30) | **R** |
| Colistin Sulphate (CT, 10) | **R** |
| Ampicillin (AMP, 10) | **S** |

*Interpretation of the inhibition zone diameters is susceptible **(S)**; resistant **(R)** according to CLSI (2017).
